# Label-Free Amperometric Immunosensor Based on Versatile Carbon Nanofibers Network Coupled with Au Nanoparticles for Aflatoxin B_1_ Detection

**DOI:** 10.3390/bios11010005

**Published:** 2020-12-24

**Authors:** Yunhong Huang, Fei Zhu, Jinhua Guan, Wei Wei, Long Zou

**Affiliations:** 1 State Key Laboratory for Managing Biotic and Chemical Threats to the Quality and Safety of Agro-Products, Institute of Agro-Product Safety and Nutrition, Zhejiang Academy of Agricultural Sciences, Hangzhou 310021, China; sallyyunhong@jxnu.edu.cn; 2 Nanchang Key Laboratory of Microbial Resources Exploitation & Utilization from Poyang Lake Wetland, College of Life Sciences, Jiangxi Normal University, Nanchang 330022, China; zhufei@jxnu.edu.cn (F.Z.); guanjh@jxnu.edu.cn (J.G.)

**Keywords:** aflatoxin B_1_, immunosensor, carbon nanofibers, Au nanoparticles, electrochemical detection

## Abstract

Facile detection methods for mycotoxins with high sensitivity are of great significance to prevent potential harm to humans. Herein, a label-free amperometric immunosensor based on a 3-D interconnected carbon nanofibers (CNFs) network coupled with well-dispersed Au nanoparticles (AuNPs) is proposed for the quantitative determination of aflatoxin B_1_ (AFB_1_) in wheat samples. In comparison to common carbon nanotubes (CNTs), the CNFs network derived from bacterial cellulose biomass possesses a unique hierarchically porous structure for fast electrolyte diffusion and a larger electrochemical active area, which increases the peak current of differential pulse voltammetry curves for an immunosensor. Combined with AuNPs that are incorporated into CNFs by using linear polyethyleneimine (PEI) as a soft template, the developed Au@PEI@CNFs-based immunosensor showed a good linear response to AFB_1_ concentrations in a wide range from 0.05 to 25 ng mL^−1^. The limit of detection was 0.027 ng mL^−1^ (S/N = 3), more than three-fold lower than that of an Au@PEI@CNTs-based sensor. The reproducibility, storage stability and selectivity of the immunosensor were proved to be satisfactory. The developed immunosensor with appropriate sensitivity and reliable accuracy can be used for the analysis of wheat samples.

## 1. Introduction

Aflatoxins, as the toxic metabolites of *Aspergillus flavus, A. nomius* and *A. parasiticus*, are well known to be severely harmful to human beings and animals since they are highly carcinogenic, teratogenic and hepatotoxic [1,2]. Across the world, many agricultural and food products such as peanut, maize, wheat, rice and soybean are naturally contaminated by aflatoxins during the periods of growth, harvest and storage [3]. Particularly, the hot and humid weather in the southwest and south of China is highly favorable to aflatoxin contamination, which has become a serious threat to human health and an economic barrier [4]. Among more than twenty identified aflatoxins, aflatoxin B_1_ (AFB_1_) is the most toxic and has been regarded as a group I carcinogen by the International Agency for Research on Cancer. Meanwhile, regulatory limits are enforced in many countries and regions for AFB_1_ levels to avoid its overexposure to human and animals. In China, the maximum allowed AFB_1_ level is as low as 5 μg kg^−1^ for many types of cereals (China National Standard No. GB 2761-2017) [5]. The European Union sets a limit of 2 μg kg^−1^ on AFB_1_ in cereals, nuts and their processed products (European Commission Regulation No. 1881/2006/EC) [6]. With the increasing concerns and the intensifying legislative framework about AFB_1_ worldwide, an efficient analytical method capable of simple, rapid and sensitive detection in food or other edibles is of crucial importance.

Currently, several chromatography-based analytical techniques, including but not limited to high-performance liquid chromatography (HPLC) [7,8,9] and liquid chromatography-tandem mass spectrometry (HPLC-MS) [10,11,12], have been developed as standard methodologies for the quantitative determination of AFB_1_ with high sensitivity and accuracy. However, these methodologies generally rely on well-equipped laboratory facilities, time-consuming sample pretreatment and skilled operators, thereby rending them ineligible for rapid screening of large amounts of actual samples. [13] Meanwhile, immunoassay-based methods, for example enzyme-linked immunosorbent assay (ELISA) [14], immunochromatographic strip (ICS) [15,16] and dot-immunochromatography flow through assay strip (DIGFA) [17], have been documented significantly for AFB_1_ detection over the past few decades owing to reliable sensitivity, excellent selectivity and suitability for high-throughput screening. In particular, electrochemical immunosensors that incorporate ultrasensitive electrochemical signals into immunological recognition depending on specific antibody-antigen interactions have attracted extensive interests. [18] For all we know, the construction of a well-defined functional electrode interface for signal amplification, together with enhanced stability, is a critical aspect for the development of electrochemical immunosensors. Functional nanomaterials such as nanostructured noble metals (e.g., gold nanoparticles, AuNPs) have powerful benefits in this direction on account of their features of high conductivity, excellent catalytic activity, etc. [19,20,21,22,23] In addition, apart from electrochemical immunosensors, AuNPs have been in other types of biosensors including, but not limited to, colorimetry [17,24,25], chemiluminescence [26], fluorescence [27] and surface plasmon resonance (SPR) [28,29] analyses because of their flexible features and high biocompatibility. Notably, pure nanoparticles commonly tend to aggregate during application, possibly leading to low practicability. As an effective approach, the incorporation of AuNPs into nanostructured carbon substrates, with large surface area and remarkable conductivity, is the most commonly used route to improve electrochemical performance. [30] Diverse aspects of carbon nanomaterials, including 0-D carbon dots [31,32], 1-D carbon nanotubes [33,34,35] and 2-D graphene [36,37], have been documented widely. Moreover, 3-D carbon nanomaterials with a hierarchical porous structure have demonstrated significant achievements in developing an effective electron exchange interface for both energy conversion and electrochemical sensing [38,39]. Such a 3-D porous nanostructure not only provides a highly interconnected electron transfer network and extraordinarily large electrochemical-active area, but also allows fast electrolyte diffusion.

In our previous work, we fabricated a novel nano carbon network consisting of carbon nanofibers (CNFs) derived from bacterial celluloses (a special biomass product from microbial synthesis) for rationally functionalizing bioelectrodes [40]. Because of its unique 3-D hierarchically porous structure, constructed by cross-linked nanofibers with a diameter of 10–30 nm, and excellent biocompatibility, the bio-abiotic interfacial electron exchange improved dramatically when used to wire biological molecules onto electrodes, thereby leading to both high-powered bioelectricity production in microbial fuel cells and high-sensitive enzyme sensors [40,41]. Taken together, the bacterial celluloses-derived CNFs could be promising candidates to tailor an electrode interface for high-performance biosensors on account of their 3-D porous structure and superior biocompatibility. Herein, we attempt to develop a simple, sensitive and stable electrochemical immunosensor based on an AuNP-decorated CNFs network for rapid detection of AFB_1_. The hybrid electrode material was synthesized through the in-situ growth of AuNPs on interconnected carbon nanofibers with the help of linear polyethyleneimine (PEI) as a soft template according to a previous report [42], and its electrochemical properties were investigated carefully in comparison to AuNPs-modified CNTs as a control. Afterwards, an amperometric immunosensor for quantifying AFB_1_ was established and optimized, then confirmed by recovery in spiked wheat samples. This work is expected to further illustrate the great practical value of CNFs network material in biologically hybridized electrochemical systems and provide an ingenious route to construct a robust electrochemical electrode for biosensing.

## 2. Materials and Methods

### 2.1. Chemicals and Reagents

AFB_1_ (purity ≥98.0%), aflatoxin B_2_ (AFB_2_, purity ≥ 98.0%), aflatoxin G_1_ (AFG_1_, purity ≥ 98.0%), aflatoxin G_2_ (AFG_2_, purity ≥ 98.0%), ochratoxin A (OTA, purity ≥ 98.0%), deoxynivalenol (DON, purity ≥ 98.0%), zearalenone (ZEN, purity ≥ 98.0%), bovine serum albumin (BSA, purity ≥ 98.0%), chloroauric acid (HAuCl_4_·3H_2_O, purity ≥ 99.9%), and PEI (average M.W. 2500, purity ≥ 99.0%) were purchased from Sigma-Aldrich (Shanghai) Trading Co., Ltd. (Shanghai, China). Monoclonal antibody (Ab, 1 mg mL^−1^, purified by protein G resin) against AFB_1_ was provided by Dr. D. Wang. [43] The multiwalled CNTs (diameter: 20–40 nm, length: 1–2 μm, purity: > 95%) were purchased from Shenzhen Nanotech Port Co. Ltd. (Shenzhen, China), and were purified according to our previous work [44]. The hydrogel pellicles of bacterial celluloses were provided by Ms. C.Y. Zhong (Hainan Yeguo Foods Co., Ltd. Haikou, China). Wheat samples were bought from a local supermarket. All other inorganic chemicals and organic solvents were analytical reagents grade, purchased from China National Pharmaceutical Industry Co., Ltd. (Beijing, China), and used without further purification. All aqueous solutions and buffers were prepared with deionized water (resistance of 18.2 MΩ cm^−1^) produced from a Millipore Q water purification system.

### 2.2. Apparatus

Cyclic voltammetry (CV) and differential pulse voltammetry (DPV) were performed on a CHI 760E electrochemical workstation (Chenhua Instrument Shanghai Co., Ltd. Shanghai, China). A typical three-electrode system consisting of an Ag/AgCl reference electrode, a Pt wire counter electrode and a modified glassy carbon electrode (GCE, Φ = 3 mm) as working electrode, was used for all electrochemical measurements. The morphologies of prepared nanomaterials were observed on a JSM-7800F field emission scanning electron microscope (FESEM, JEOL, Tokyo, Japan) and a JEM-2100F transmission electron microscope (TEM, JEOL, Tokyo, Japan).

### 2.3. Preparation of Au@PEI@CNFs Nanocomposites

The CNF aerogels were derived from hydrogel pellicles of bacterial celluloses through vacuum freeze drying followed by carbonization at 1000 °C under a flowing argon atmosphere, according to our previous report [40]. The hybridized nanomaterials were synthesized by in-situ formation of AuNPs on PEI-functionalized CNFs under a mild heating condition, where the PEI not only acts as a dispersing agent but also a reducing agent for reduction of HAuCl_4_, as demonstrated by Hu et al. [42]. In a typical synthesis, 2.5 mg CNFs were dispersed in 5 mL of 0.3 wt% PEI aqueous solution with the help of ultrasonic processing for 2 h. After centrifugal collection, the precipitates of PEI-modified CNFs (PEI@CNFs) were resuspended in 5 mL of 1 mM HAuCl_4_ solution followed by magnetic stirring for 30 min at ambient temperature (25 ± 2 °C). The suspension was kept at 60 °C under static condition for 1 h, leading to the formation of Au/PEI/CNFs hybrids accompanied by the color change from yellowish to black-purple. The product was collected by centrifugation at 10,000 g for 10 min at 10 °C and washed with deionized water until the supernatant became colorless after centrifugation. Finally, the precipitates of Au@PEI@CNFs nanocomposites were dried at 60 °C in a vacuum dryer. The Au@PEI@CNTs used for comparison were synthesized by the same approach above, except that the CNTs replaced the CNFs.

### 2.4. Fabrication of Amperometric Immunosensor

**Figure 1:**
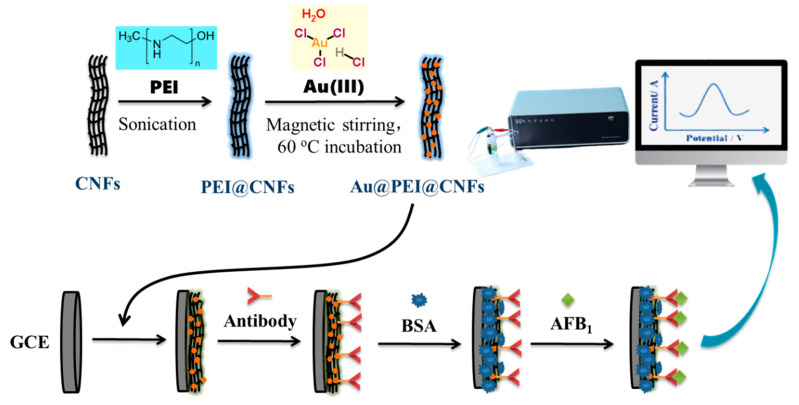
Stepwise illustration of fabrication procedure of the amperometric immunosensor based on interconnected carbon nanofibers (CNFs) network decorated with AuNPs (Au@PEI@CNFs nanocomposites).

The stepwise schematic illustration of the fabrication procedure for the AFB_1_ immunosensor is illustrated in Figure 1. The GCE was successively polished with alumina slurry of 1.0, 0.3 and 0.05 μm, followed by sonication (frequency of 20 kHz) for 30 s at 25 ± 2 °C in deionized water and ethanol in turn, and then dried at room temperature (25 ± 2 °C). A sample of 10 μL of the Au/PEI/CNFs suspension (1 mg mL^−1^) was dropped on the electrode surface, followed by evaporation of the solvent in air. Afterwards, 6 μL of anti-AFB_1_ antibody in 0.01 M phosphate-buffered saline (PBS, pH 7.0) was immobilized onto the modified electrode through electrostatic interaction during an incubation process at 37 °C under a moisturizing condition. After washing with PBS, the electrode was kept in a 3 wt% BSA solution for 1 h to eliminate nonspecific binding sites and then rinsed. Then 6 μL of AFB_1_ solution with a series of concentrations (0.05, 0.2, 1, 2, 5, 10, 15, 20 and 25 ng mL^−1^) was dropped onto the electrode surface for specific combination for a certain amount of time. After being rinsed with PBS containing 0.05% Tween-20 and dried, the assembled immunosensor was stored at 4 °C for subsequent use.

### 2.5. Sample Preparation

Noncontaminated wheat samples obtained from a local market, which were verified by a commercial ELISA kit with a limit of detection (LOD) of 0.1 μg kg^-1^ (Huaan Magnech Bio-tech Co., Ltd., Beijing, China), were ground. Aliquots (5 g) of pulverized samples were extracted with 25 mL methanol–water (70:30, *v*/*v*) for 30 min on a vortex shaker. After centrifugation at 10,000 g for 10 min at 10 °C, the supernatant was filtered through a 0.45μm filter membrane (Jinteng Experimental Equipment Co., Ltd., Tianjin, China) and then stored at −20 °C for further assay. The extracting solution was diluted five-fold with 0.01 M PBS buffer before AFB_1_ detection, which means a total dilution of 25-fold. In order to evaluate accuracy and precision of the developed immunosensor, three spiked wheat samples with AFB_1_ concentrations of 5, 25 and 100 μg kg^−1^ were prepared, followed by being extracted according to above approach for determination of AFB_1_.

### 2.6. Electrochemical Measurements

The modified electrodes were characterized using CV at a scan rate of 30 mV s^−1^ by scanning the potential from −0.2 to 0.6 V, which was performed in a deaerated PBS solution containing 5 mM K_3_[Fe(CN)_6_]/K_4_[Fe(CN)_6_] as a redox probe. DPV for AFB_1_ determination was recorded with a potential ranging from −0.2 to 0.6 V, pulse amplitude of 50 mV, pulse width of 0.05 s and pulse period of 0.5 s. DPV peak current change (ΔIp) was calculated by using the equation of ΔI = I_0_ − I_i_, where I_0_ was the current response obtained in blank solution and I_i_ was the current response obtained in the case of AFB_1_ concentrations.

## 3. Results and Discussion

### 3.1. Characterization of Nanocomposites

**Figure 2:**
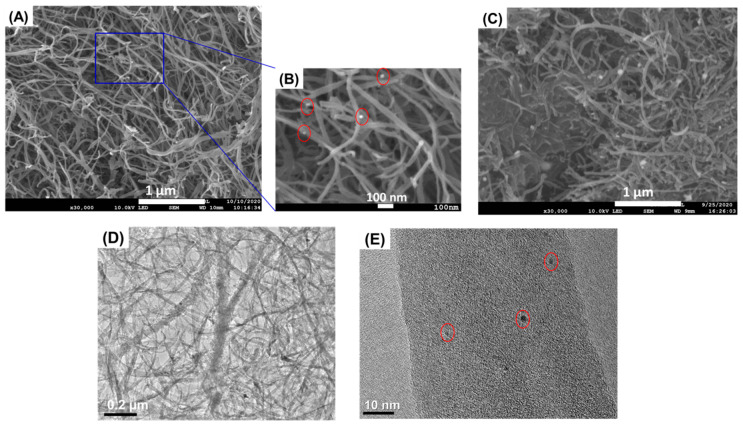
Morphological characterization of the prepared nanocomposites. SEM images of Au@PEI@CNFs (**A**,**B**) and Au@PEI@CNTs (**C**), TEM images of Au@PEI@CNFs (**D**,**E**).

**Figure 3:**
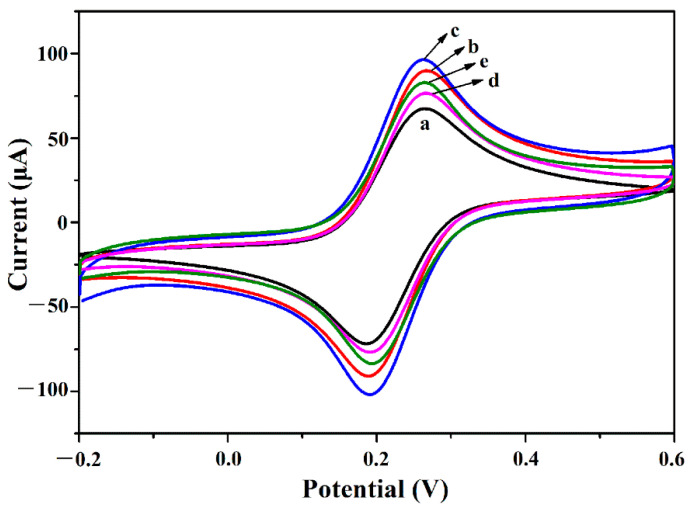
Electrochemical characterization of the prepared nanocomposites. CV graphs of (line a) bare glass carbon electrode (GCE), (line b) GCE/CNFs, (line c) GCE/Au@PEI@CNFs, (line d) GCE/carbon nanotubes (CNTs) and (line e) GCE/Au@PEI@CNTs, respectively. CVs were recorded at a scan rate of 30 mV s^−1^ in PBS solution containing 5 mM K_3_[Fe(CN)_6_]/K_4_[Fe(CN)_6_].

Surface morphologies of the as-prepared Au@PEI@CNFs and Au@PET@CNTs nanocomposites were observed by FESEM. The Au@PEI@CNFs (Figure 2A,B) showed an interconnected network architecture consisting of carbon nanofibers with a diameter range from 20 to 50 nm. In spite of undergoing the chemical modification process, the 3-D structure of CNFs [40] remained intact, indicating its plasticity and toughness. On the contrary, the Au@PET@CNTs (Figure 2C) exhibited a dense structure due to aggregation. Intuitively, this stuffed structure would be not conducive to diffusion of electrolyte and interfacial electrochemical reaction compared to the 3-D reticular porous architecture of Au@PEI@CNFs. AuNPs with a size range from several to dozens of nanometers were, as expected, grafted uniformly on the surfaces of both CNFs and CNTs because of the dispersion effect and reduction action of the PEI coating. The TEM image (Figure 2D) of Au@PEI@CNFs further demonstrated the cross-linked structure of CNFs and the depositions of AuNPs. In addition, some small nanocrystals were also observed on the fibers of CNFs in the high-resolution TEM image (Figure 2E), which were deemed to be nucleation sites of AuNPs. Subsequently, the as-prepared powdered materials were dropped onto a GCE surface for electrochemical characterization. As shown in Figure 3, the CV curves of all tested electrode materials showed a couple of well-defined and reversible redox peaks originating from the redox reaction of [Fe(CN)_6_]^3−/4−^ on the electrode interfaces, and an increased peak-current in varying degrees in comparison to the bare GCE. Moreover, according to the peak area of the CV curve, the estimated electrochemical active area of Au@PEI@CNFs was approximately 1.23-fold that of Au@PEI@CNTs and of CNFs was 1.27-fold, which could be mainly attributed to the much larger surface area and more accessible pores of CNFs than the CNTs [40]. Taken together, the CNFs could be demonstrated to be an applicable substrate for constructing functional nanomaterials with efficient 3-D reticular porous structures and promising electrocatalytic performances.

### 3.2. Immunosensor Fabrication

**Figure 4:**
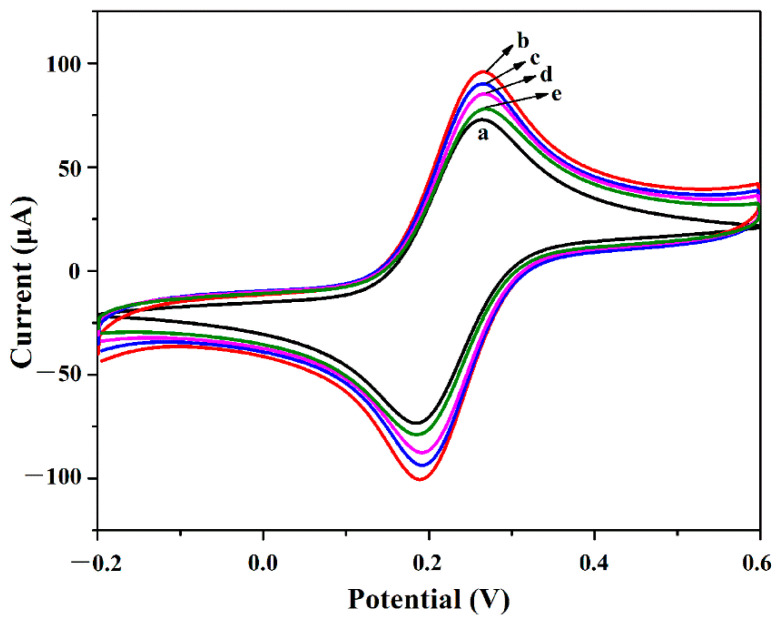
Electrochemical monitor of the immunosensor fabrication. CV curves of (line **a**) GCE, (line **b**) GCE/Au@PEI@CNFs, (line **c**) GCE/Au@PEI@CNFs/Ab, (line **d**) GCE/Au@PEI@CNFs/Ab/BSA and (line **e**) GCE/Au@PEI@CNFs/Ab/BSA/AFB_1_ in PBS solution (pH 7.4) containing 5 mM K_3_[Fe(CN)_6_]/K_4_[Fe(CN)_6_]. Scan rate: 30 mV s^−1^.

The fabrication process of the immunosensor is another key issue for the development of sensing platform, which was monitored using a CV technique after each step [45]. As shown in Figure 4 (line a), there were well-defined and reversible [Fe(CN)_6_]^3−/4−^ redox peaks for the bare GCE. The peak-current of the Au@PEI@CNFs nanocomposites modified GCE as shown in Figure 4 (line b) increased remarkably, which could be probably attributed to the high conductivity and large electrochemical active area of the Au@PEI@CNFs nanocomposites. While the peak-current decreased apparently after being modified with anti-AFB_1_ antibody, as shown in Figure 4 (line c). Such attenuation of peak-current indicated that the anti-AFB_1_ antibody was successfully immobilized on the electrode surface because the nonconductive molecule can retard the interfacial electron transfer between the redox probe of [Fe(CN)_6_]^3−/4−^ and the electrode surface. Then, the as-prepared electrode was immersed in BSA solution to block any possible remaining nonspecific active sites, which resulted in a further decrease of peak-current for the same reason. More importantly, the peak-current decreased again after the AFB_1_ was dropped onto the electrode, which indicated the successful capture of AFB_1_ and the formation of an immunocomplex after the specific recognition between antigen and antibody. Such a produced immunocomplex layer greatly hindered interfacial electron transfer. Therefore, the fabricated GCE/Au@PEI@CNFs/Ab/BSA/AFB_1_ immunosensor can be used for the detection of AFB_1_.

### 3.3. Optimization of Determination Conditions

**Figure 5:**
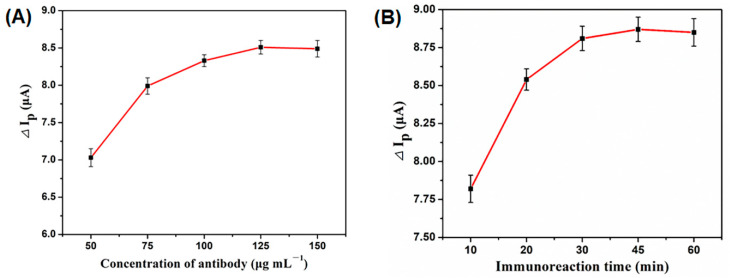
Effects of the antibody concentration (**A**) and immunoreaction time (**B**) on the detection sensitivity of the proposed immunosensor, error bar = RSD (n = 3).

The sensitivity of an immunosensor relies, o a large extent, on the concentration of antibody and the immunoreaction time [46,47]. The effects of these two influential factors were examined by means of DPV, and the peak-current decrease (ΔIp) was applied as the indicator to evaluate the optimum condition for sensitively detecting the target analyte of 5 ng mL^−1^ AFB_1_. As shown in Figure 5A, the ΔIp increased gradually with increasing concentration of antibody (too low an amount of antibody could not recognize the target analyte effectively), and then tended to be stable at 125 μg mL^−1^. In consequence, an antibody concentration of 125 μg mL^−1^ was selected for the preparation of the proposed immunosensor. Likewise, the ΔIp increased gradually with increasing immunoreaction time from 10 to 45 min and decreased slightly with a further increase in time (Figure 5B). Therefore, an immunoreaction time of 45 min was adopted as the optimal antigen/antibody binding time throughout the subsequent experiment.

### 3.4. Analytical Performance of the Immunosensor

**Figure 6:**
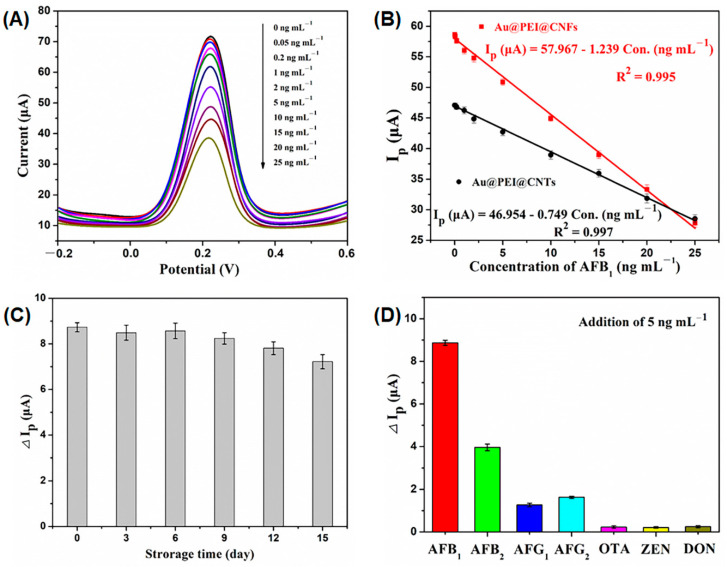
Analytical performance of the proposed immunosensor. (**A**) Differential pulse voltammetry (DPV) responses of the Au@PEI@CNFs-based immunosensor to different concentrations of AFB_1_. (**B**) Calibration curves of immunosensors to different concentrations of AFB_1_, error bar = RSD (n = 6). (**C**) Peak-current change (ΔIp) responses of immunosensors to different storage time, error bar = RSD (n = 3). (**D**) Peak-current change (ΔIp) responses of immunosensors to different mycotoxins (5 ng mL^−1^), error bar = RSD (n = 3).

The analytical performance of the proposed immunosensor was carried out by determination of different AFB_1_ concentrations by means of DPV under the optimized conditions. Firstly, the nonspecific adsorption of AFB_1_ on Au@PEI@CNFs-based immunosensor was evaluated through the decrease in peak-current when in the absence of anti-AFB_1_ antibody. Only a 0.45 μA of attenuation was detected after the addition of 5 ng mL^−1^ AFB_1_, which was about 5% of that when in the presence of anti-AFB1 antibody. This indicated that the decrease in peak-current for the Au@PEI@CNFs-based immunosensor was almost entirely attributed to the specific capture of AFB_1_ by the antibody and the nonspecific adsorption had a negligible impact on the analytical capacity. Apparently, as shown in Figure 6A, the peak-current (Ip) decreased gradually with increasing concentration of AFB_1_, which indicated the workable capacity of quantitative analysis for the immunosensor. The relationship of the Ip and the concentration of AFB_1_ was well fitted into a simple linear regression in a range from 0.05 to 25 ng mL^−1^ (Figure 6B). For the Au@PEI@CNFs functionalized immunosensor, the linear regression equation of the calibration curve was Ip (μA) = 57.967–1.239 Con. (ng mL^−1^) with a determination coefficient (*R*^2^) of 0.995 (Con. is the abbreviation for concentration). The LOD was estimated to be 0.027 ng mL^−1^ (S/N = 3, n = 6), much lower than that for the Au@PEI@CNTs-based immunosensor (0.093 ng mL^−1^). Apparently, the low LOD of developed immunosensor based on Au@PEI@CNTs was enough to meet the limit standards of AFB_1_ prescribed by China and European Commission in the majority of agricultural and food products (5 and 2 μg kg^−1^, respectively) and even infant food (0.5 and 0.1 μg kg^−1^, respectively). Given the consistent immunosensor fabrication approach and analytic conditions, the superior detection sensitivity for the Au@PEI@CNFs electrode material could be due to its particular 3-D reticular porous structure and large electrochemical active area. In addition, the dynamic range and LOD of this proposed immunosensor were comparable to most previous reports on the amperometric immunosensors (Table 1).

The reproducibility, storage stability and selectivity of the immunosensor were investigated subsequently. A series of six immunosensors based on Au@PEI@CNFs nanocomposites were prepared in the same way to detect AFB_1_ (5 ng mL^−1^). The relative standard deviation (RSD) of the measurements for the six immunosensors was 4.66%, which suggested that the reproducibility of the proposed immunosensor for AFB_1_ detection was acceptable. In order to evaluate storage stability, three immunosensors were fabricated and stored at 4 °C under refrigeration. These immunosensors were applied for the determination of AFB_1_, and the electrochemical responses of them were recorded every three days (Figure 6C). After three and nine days, respectively, 97.25 and 94.39% of initial ΔIp remained. Nevertheless, after twelve days less than 90% of the initial ΔIp was observed. Taken together, the stability of the immunosensor was richly satisfactory within one week, which could result from good stability of both antibody and Au@PEI@CNFs nanocomposites, as well as their form immobilization on the GCE surface. Further, the cocontamination of other mycotoxins (including the homologues of AFB_1_ as well as DON, OTA and ZEN) often occurs in wheats and their products. [48] Therefore, the cross-reactivity of the developed immunosensor towards these mycotoxins was investigated (Figure 6D). Expectedly, the cross-reactivities for all tested mycotoxins, except for the homologues of AFB_1_ (i.e., AFB_2_, AFG_1_ and AFG_2_), were less than 5% because the specificity of an immunosensor is primarily dependent on that of the used antibody, while the cross-reactivities for AFB_2_, AFG_1_ and AFG_2_ were 44.64, 14.32 and 18.38%, respectively. This result indicated that the developed immunosensors was suitable to detect AFB_1_ rather than the total aflatoxins, which is mainly dependent on the affinity and specificity of the monoclonal antibody used.

### 3.5. Analysis of Wheat Samples

As indicated above, the proposed label-free amperometric immunosensor showed great application potential in AFB_1_ determination. For the sake of proving the feasibility for real sample analysis, the as-prepared immunosensors were used to detect the blank wheat samples spiked with standard AFB_1_ at different concentrations (2, 25, and 200 µg kg^−1^). The extracts of the spiked samples were diluted five-fold before undergoing electrochemical analysis to mitigate sample matrix interference as much as possible. The recovery values of intra-assay and interassay lay in the range of 89.06–105.80% and 85.94–111.60%, respectively (Table 2). Correspondingly, the RSD values were in the range of 7.38–11.35% and 10.04–13.80%, respectively. Apparently, all test values of both recovery and RSD were within the corresponding reference ranges for a quantitative method prescribed by European Commission (Decision No. 2002/657/EC) [56], except for the inter-assay recovery of 111.60% at the low spiked concentration (5 µg kg^−1^), slightly exceeding the recommended maximum reference value of 110%. There was a tendency of the recovery value to decrease as the spiked concentration of AFB_1_ increased, which might be due to the incomplete extraction at the high concentration, but this would not affect the immunosensor used to determine whether a wheat sample is contaminated or not. Overall, the results suggested that the developed immunosensor could be applied to detect AFB_1_ in real samples with acceptable accuracy and precision.

## 4. Conclusions

In this work, a CNFs network was successfully adopted to a 3-D carbonaceous substrate for constructing functional nanocomposites to improve the performance of an immunosensor. On account of its unique interconnected structure with hierarchical pores and large surface area, the CNFs network showed superior electrochemical features compared to common CNTs when combined with AuNPs. In consequence, the Au@PEI@CNFs-based immunosensor achieved a wide dynamic range from 0.05 to 25 ng mL^−1^ with a low LOD of 0.027 ng mL^−1^, which was three-fold more sensitive than the Au@PEI@CNTs-based one. The immunosensor also exhibited excellent stability and selectivity, and good applicability in analysis of real wheat samples. Thus, the CNFs network has been proved substantially to be a promising functional nanomaterial for construction of biosensors based on amperometric analysis. In addition, its feasible application in electrochemical impedance spectroscopy-based biosensors would be another interesting research topic in view of a possible higher detection sensitivity, requiring a further specific study.

## Figures and Tables

**Table 1:** Comparison of the proposed immunosensor and other amperometric sensors.

Modified Electrode	Linearity (ng mL^−1^)	LOD (ng mL^−1^)	Reference
Au@PEI@CNFs/GCE	0.05–25	0.027	This work
Au@PEI@CNTs/GCE	0.05–25	0.093	This work
CNTs/PDDA/Pd-Au/GCE	0.05–25	0.03	[34]
AuNPs/PEDOT-GO/GCE	0.5–60	0.109	[49]
CHI-AuNPs/GCE	0.2–30	0.12	[50]
CHI-AuNPs/GCE	0.1–30	0.06	[51]
Au/PANI/GN/GCE	0.05–25	0.034	[52]
PTH/AuNP/GCE	0.6–2.4	0.07	[53]
Au/TiO2/RTIL/Nafion/GCE	0.1–12	0.050	[54]
MWCNTs/AFO	1–225	0.5	[55]
PoPD/3DNEEs	0.04–8	0.019	[20]

**Table 2:** Recovery analysis of AFB_1_ from spiked wheat samples by the proposed immunosensor.

Spiked Concentration (µg kg^−1^)	Detected Concentration (Mean ± SD, µg kg^−1^)	Recovery (%)	RSD (%)
	Intra-assay (n = 6)		
5	5.29 ± 0.61	105.80	11.35
25	23.58 ± 2.16	94.32	9.16
200	178.13 ± 13.15	89.06	7.38
	Inter-assay (n = 6)		
5	5.58 ± 0.77	111.60	13.80
25	24.43 ± 3.02	97.72	12.36
200	171.88 ± 17.26	85.94	10.04

## Data Availability

Not applicable.
